# Predictive values of ultrasound characters associated with malignant thyroid nodules in Yaoundé: a cross-sectional study

**DOI:** 10.11604/pamj.2024.47.38.42190

**Published:** 2024-01-30

**Authors:** Yannick Mossus, Roger Christian Meva'a Biouélé, Leonel Christophe Atanga, Adèle-Rose Ngo Nyeki, David Mindja Eko, Olive Nicole Ngaba Mambo Pouka, François Djomou, Louis Richard Njock, Alexis Ndjolo

**Affiliations:** 1 Department of Ophthalmology, Ear Nose and Throat (ENT) and Stomatology, Faculty of Medicine and Biomedical Sciences, University of Yaoundé I, Yaoundé, Cameroon

**Keywords:** Ultrasound characters, predictive values, malignant thyroid nodules, cross-sectional study, Yaoundé

## Abstract

**Introduction:**

most ultrasound criteria are defined in developed countries and commonly used in practice to assess the malignancy risk of thyroid nodules. This practice does not take into consideration some aspects of our context as delay of consultation and insufficient iodine intake. The objective of this study was to determine the predictive values of ultrasound characters associated with malignant thyroid nodules in our environment.

**Methods:**

we conducted a cross-sectional, prospective, and analytical study in three hospitals in Yaoundé over a six-month period in 2022. Our sample consisted of thyroid nodules with ultrasound, cytopathological, and histopathological data. The ultrasound characters and histology status of category III thyroid nodules and higher in Bethesda score were analysed in univariate and multivariate statistics to determine their predictive values.

**Results:**

eighty-nine nodules were obtained according to our inclusion criteria. The sex ratio was 0.46 and the average age of the patients was 46 years (IQR=42-59). The cancer prevalence in our sample was 22.47%. On ultrasound assessment, the characters associated to malignant histology (p<0.05) were nodules count, echogenicity, echostructure, presence or absence of microcalcifications, margins, and type of vascularization. Positive predictive values ranged from 26.15 to 57.14%, while negative predictive values ranged from 12.5 to 33.3%.

**Conclusion:**

taken alone, the ultrasound characters of suspected thyroid nodules have poor predictive values. There was a high variability in sensitivity but that was generally good (60-95%) while specificity was low. The prediction of malignant thyroid nodules is correlated with the association of at least two ultrasound criteria supported by clinical arguments.

## Introduction

The thyroid nodule is any localized hypertrophy of the thyroid gland [1]. In countries with sufficient iodine intakes, the clinical prevalence is 5.3 and 6.4% for women, 0.8 and 1.6% for men. The prevalence is about 3 times higher in women and increases with age [1,2]. The ultrasound prevalence is about 10 times higher, roughly equal to that of the decade of the subjects examined [1,2]. In Cameroon, the ultrasound prevalence of thyroid nodules is 28.3% according to Moifo *et al*. [3]. On ultrasound, several characters are used to describe the thyroid nodule: size, shape, margins, echogenicity, echostructure, type of vascularization, and capsule break-in. Other associated lesions such as the presence or absence of microcalcifications in the thyroid parenchyma may help to histological diagnosis [1].

The Thyroid Imaging Reporting and Data System (TIRADS) classifies thyroid nodules according to their malignancy risk using ultrasound criteria [3,4]. However, several TIRADS classifications exist according to world regions [4-9]. It is therefore apparent that a highly reliable, reproducible, and clinically practical TIRADS classification will greatly improve communication between clinicians and radiologists. This will even be more helpful in settings where Fine Needle Aspiration Biopsy (FNAB) is not readily available and so decisions will therefore be based to a great extent on the ultrasound features of the lesions and TIRADS classification as this implies the potential risk for malignancy [3]. In the absence of studies reporting predictive values of nodule-related ultrasound characters in practice in sub-Saharan Africa, radiologists and clinicians use the different TIRADS classifications (Eu-TIRADS and ACR-TIRADS) to characterize nodules on ultrasound. The aim of our study was to determine ultrasound characters associated with malignant thyroid nodules and their predictive values in Yaoundé.

## Methods

**Study design and setting:** we conducted a cross-sectional, prospective, and analytical study during a six-months period (April to September 2022) in three university hospitals in Yaoundé.

**Participants:** our sampling was not exhaustive. The source population consisted of patients who underwent thyroid surgery and the target population was patients with thyroid nodules on ultrasound and whose cytological examination of the echoguided FNAB showed a category III or higher according to the Bethesda´s score, edition 2017 [10]. We excluded patients whose ultrasound did not provide all the descriptive features of thyroid nodules and those whose histology was not recorded in the file.

**Variables:** variables in ultrasound included nodules count, size, shape, margins, echogenicity, echostructure, type of vascularization, capsular break-in or not, presence of microcalcifications or not [1]. In histology, histopathological status was our main variable of interest.

**Data sources:** data were collected through a structured questionnaire and reported in a database. Nodules count, size, shape, margins, echogenicity, echostructure, type of vascularization, capsular break-in or not, and presence of microcalcifications or not were assessed in ultrasound. The histological status of nodules was given by anatomopathologist.

**Bias:** to exclude any potential bias, we asked to ultrasonographist to describe nodules in the same way.

**Study size:** according to an ultrasound prevalence of thyroid nodules at 28.3% [3], an accuracy of 1.5%, and a type I error of 5%, we obtained a sample size of 312 nodules by using OpenEpi software.

**Quantitative variables:** quantitative data were analyzed by grouping them by interval. Groups were done according to the literature for better comparison. The age was split in 10-year intervals and the nodule size in <7 mm, 7-20 mm, and >20 mm.

**Statistical methods:** collected data were analysed using the r-studio software for Windows version 4.3.1. Qualitative data were represented by numbers and frequencies. Continuous variables were expressed by their mean (standard deviation) when normally distributed; otherwise, by their median (interquartile interval (IQR)). The ultrasound characters of the nodules were opposed to malignant nodules to determine the association link. The Chi-square test or Fisher´s exact probability was used for the proportion comparison. Continuous variables were compared using the t-student test or its nonparametric equivalent. Logistic regression was used to investigate independent factors associated with malignancy. The explanatory variables associated with malignancy in univariate analysis with p<0.05 were introduced in the same logistic regression model to look for independent factors associated with malignant nodules. The significance level was p<0.05. Predictive values, sensitivity, and specificity were calculated for each ultrasound character frequently associated with malignant nodules and reported in the literature. These were the oval shape, size >7 mm, hypoechogenicity, solid echostructure, irregular margins, and mixed vascularization.

**Ethical considerations:** permissions to carry out the study was obtained from the *Comité Institutionnel d´Ethique et de Recherche* of the Faculty of Medicine and Biomedical Sciences at the University of Yaoundé I and from General Managers of Hospitals. Written informed consent was obtained from the study participants before their surgery. This consent includes the possibility to use their informations for scientific purposes.

## Results

**Participants:** the surgery procedure was performed on 112 patients for category III thyroid nodules and higher in Bethesda´s score. We excluded 16 for incomplete data on thyroid ultrasound and 7 for lack of post-operative histology. Thus, our study was based on a sample of 89 thyroid nodules.

**Descriptive data:** women made up 69% (n=61) of our sample with a sex ratio of 0.46. The median age was 46 years (IQR=42-59) with extremes of 16 and 72 years (Table 1).

**Table 1:** sex and age of patients

Variables and modalities (n=89)	n (%)
**Sex**	
Male	28 (31%)
Female	61 (69%)
**Age**	
≤30	18 (20%)
(30,40)	19 (21%)
(40,50)	20 (22%)
(50,60)	12 (13%)
(60,70)	16 (18%)
>70	4 (4.5%)

Post-operative histopathological analysis found 22.47% of malignant nodules (n=20) against 77.53% (n=69) of benign nodules. Table 2 gives the ultrasound characters of thyroid nodules and bivariate analysis according to the histological status of nodules. In ultrasound, thyroid nodules were generally multiple (69%), ranging in size from 7 to 20 mm (63%), oval (72%), hypoechogenic (63%), solid echostructure (69%), irregular margins (56%) and without capsular break-in (79%). Their vascularization was either mixed (38%) or central (40%) and microcalcifications were absent in 73% of cases.

**Table 2:** ultrasound characters and bivariate analysis

Ultrasound characteristics	Total n (%)	Benign, n = 69^1^	Cancer, n = 20^1^	p-value^2^
**Nodule count**				**0.043**
Solitary	28 (31%)	18 (20.22%)	10 (11.24%)	
Multiple	61 (69%)	51 (57.30%)	10 (11.24%)	
**Size (mm)**				**0.2**
< 7	6 (6.7%)	3 (3.37%)	3 (3.37%)	
(7-20)	56 (63%)	45 (50.56%)	11 (12.36%)	
>20	27 (30%)	21 (23.60%)	6 (6.74%)	
**Shape**				**0.4**
Oval	64 (72%)	48 (53.93%)	16 (17.98%)	
Round	25 (28%)	21 (23.60%)	4 (4.49%)	
**Echogenicity**				**0.008**
Anechoic	3 (3.4%)	3 (3.37%)	0 (0.00%)	
Hypoechogenic	56 (63%)	39 (43.82%)	17 (19.10%)	
Isoechogenic	9 (10%)	6 (6.74%)	3 (3.37%)	
Hyperechogenic	21 (23.6%)	21 (23.60%)	0 (0.00%)	
**Echostructure**				**0.039**
Solid	61 (69%)	42 (47.19%)	19 (21.35%)	
Cystic	9 (10%)	9 (10.11%)	0 (0.00%)	
Mixed	13 (15%)	12 (13.48%)	1 (1.12%)	
Spongiform	6 (6,7%)	6 (6.74%)	0 (0.00%)	
**Microcalcifications**				**<0.001**
Absent	65 (73%)	57 (64.04%)	8 (8.99%)	
Present	24 (27%)	12 (13.48%)	12 (13.48%)	
**Margins**				**<0.001**
Regular	27 (30%)	27 (30.34%)	0 (0.00%)	
Irregular	50 (56%)	30 (33.71%)	20 (22.47%)	
Unclear	6 (6.7%)	6 (6.74%)	0 (0.00%)	
Clear	6 (6.7%)	6 (6.74%)	0 (0.00%)	
**Capsular break-in**				**0.12**
Present	19 (21%)	12 (13.48%)	7 (7.87%)	
Absent	70 (79%)	57 (64.04%)	13 (14.61%)	
**Type of vascularization**				**0.012**
Central	36 (40%)	33 (37.08%)	3 (3.37%)	
Peripheral	15 (17%)	12 (13.48%)	3 (3.37%)	
Mixed	38 (43%)	24 (26.97%)	14 (15.73%)	

1 : n (%); ^2^: Pearson's Chi-squared test; Fisher's exact test

**Outcome data:** the ultrasound characters associated with a malignant character (p<0.05) were nodule count, echogenicity, echostructure, presence or absence of microcalcifications, margins and type of vascularization. Associations between the malignancy of thyroid nodules and the number of nodules (OR=2.83, CI95=1.01-8.05), echogenicity (OR=2.23, CI95=1.14-5.33), echostructure (OR=3.59, CI95=1.44-18.44), mixed vascularization (OR=0.40, CI95=0.19-0.73) and microcalcifications (OR=7.12, CI95=2.45-22.09) were significant associations (p<0.05) as presented in Table 3.

**Table 3:** association strength of ultrasound characters with malignant nodule

Ultrasound characteristics	OR^1^	^2^CI95%	p-value
Nodule count	2.83	1.01-8.05	0.047
Echogenicity	2.23	1.14-5.33	0.036
Echostructure	3.59	1.44-18.44	0.033
Microcalcifications	7.12	2.45-22.09	<0.001
Margins	0.82	0.45-1.54	0.519
Intranodular vascularisation	0.40	0.19-0.73	0.006

1 OR: odds ratio; ^2^CI: confidence interval

**Main results:** predictive values of ultrasound factors associated with thyroid nodule malignancy are given in Table 4. Positive predictive values on ultrasound ranged from 26.15 to 57.14%, while negative predictive values ranged from 12.5 to 33.3%.

**Table 4:** predictive values of ultrasound factors associated with thyroid nodule malignancy

Variables	Modality	Histology		Se	Sp	PPV	NPV
		Malignant	Benign				
Shape	Oval	16	43	80	18.6	27.11	33.33
	No oval	4	8				
Size (s)	s<7 mm	17	48	85	5.88	26.15	50
	≤7 mm	3	3				
Echogenicity	Hypoechogenic	17	30	85	41.17	36.17	12.5
	Other	3	21				
Echostructure	Solid	19	36	95	29.41	34.54	6.25
	Other	1	15				
Margins	Irregular	13	27	65	47.05	32.5	22.58
	Other	7	24				
Vascularization	Mixed	14	24	70	52.94	36.84	18.18
	Other	6	27				
Microcalcifications	Present	12	9	60	82.85	57.14	16
	Absent	8	42				

Se: sensitivity; Sp: specificity; PPV: positive predictive value; NPV: negative predictive value

**Sensitivity and specificity of ultrasound characters of thyroid nodules:** sensitivity ranged from 60 to 95% and specificity 5.88 to 82.85 (Table 4).

## Discussion

Our study revealed that the number of nodules, echogenicity, echostructure, mixed vascularization, and microcalcifications were significantly associated with thyroid nodules malignancy (p<0.05). Positive predictive values on ultrasound ranged from 26.15 to 57.14%, while negative predictive values ranged from 12.5 to 33.3%. The main limitation of our study was the sample size; limited to 89 thyroid nodules.

**Sex and age of patients:** the nodular pathology of the thyroid gland predominates in female subjects with a sex ratio of 1 man to 3 to 4 women according to the series. Its incidence increases with age [2,11]. Our findings were similar to the literature with subjects mostly female (1 man for 2.18 women) beyond 40 years (57.5%).

**Nodule histology:** the risk of malignancy in front of a thyroid nodule increases with the Bethesda category. It ranges from 6-18% for category III to 94-96% for category VI. This risk is even higher in the case of non-invasive follicular thyroid neoplasia with papillary nuclear characters identical to those of carcinoma [10]. The risk of malignancy in the different Bethesda categories studied (category III to VI) was not determined in our study. On average, 5% of thyroid nodules are malignant, with rates ranging from 1.5% to 38.1% depending on patient selection [12,13]. We report a prevalence of 22.47% based on nodules selected in category III and more than Bethesda and therefore potentially malignant.

**Ultrasound characters associated with malignant thyroid nodules and association strength:** in the literature, several authors report among the ultrasound characters associated with malignant thyroid nodules those that can be considered major. Thus, Hong *et al*. in 2010 and Moifo *et al*. in 2013 identified irregular margins, taller-than-wide shape, presence of microcalcifications, and marked hypoechogenicity as major ultrasound characters associated with malignant nodules [3,14,15]. Other ultrasound characters such as lymphadenopathy and local invasion of adjacent structures are associated with nodule malignancy but are rarely found [16]. In our study, the US characters associated with the malignant nodule were number of nodules (p=0.043), echogenicity (p=0.008), echostructure (p=0.039), microcalcifications (p<0.001), margins (p<0.001), and type of vascularization (p=0.012). Capsular break-in and nodule shape are recognized as characters associated with malignant histological forms [16] but in our study, this was not the case; certainly because of the small size of our sample. After analysis of the operative parts and comparison with the ultrasound data, the number of nodules (OR=2.83, CI95=1.01-8.05), echogenicity (OR=2.23; CI95=1.14-5.33), echostructure (OR=3.59; CI95=1.44-18.44), mixed vascularization (OR=0.40; CI95=0.19-0.73) and microcalcifications (OR=7.12; CI95=2.45-22.09) had a significant association (p<0.05) with nodules in our series.

**Predictive values of ultrasound characters associated with malignant thyroid nodules:** the predictive values of the different ultrasound characters were generally poor in our study and very variable according to other series in the literature (Table 5).

**Table 5:** positive predictive values of ultrasound characters in some studies

Ultrasound characters	Our study n=89	Nam-Goong *et al*. n=317	Eisuke Koike *et al*. n=148	Moifo *et al*. n=430
Oval nodule	27.11	-	58.8	100
Size of nodule (s)	26.15 (s>7mm)	-	-	-
Solid nodule	34.54	25.6	81.8	-
Hypoechogenic nodule	36.17	27	51.4	60
Irregular margins	32.5	-	56.1	80
Mixed vascularization	36.84	-	-	-
Presence of microcalcifications	57.14	39	31.8	58.3

Each of these variables alone gives the nodule an increased risk of malignancy by a factor of 1.5 to 3 [1]. However, when these variables are associated, the risk increases significantly [17]. The solid nodule associated with other characters is the most studied association in the literature [17,18]. The size of the nodules was not predictive of malignancy in our series as in that of Besbes *et al*. in 2007 [13] but remains a prognostic element in case of malignancy if it is greater than 30 mm [1,19]. Intranodular vascularization associated or not with peripheral Doppler vascularization signals neoplastic nodular pathology [13,20]. Microcalcifications correspond to calcospherite lesions in papillary and medullary cancers but are not pathognomonic [21] despite good specificity; 82.5% in our series, 95.2% for Solbiati *et al*. [22], and 91% for Chatti *et al*. [23].

## Conclusion

Taken alone, the ultrasound characters of suspected thyroid nodules have poor predictive values. There is a high variability in sensitivity but that is generally good (60-95%) while specificity was low. The prediction of malignant thyroid nodules is correlated with the association of at least 2 ultrasound criteria supported by clinical arguments. According to the literature, the association of solid-oval-hypoechogenic-nodule with irregular contours confers a strong suspicion of malignancy to the nodule.

### What is known about this topic

- *Ultrasound characters of thyroid nodules;*
- *Predictive values of the most associated ultrasound feature with thyroid nodules over the world*.

### What this study adds

- *Predictive values of ultrasound characters associated to malignant thyroid in our practice setting;*
- *Information to take into consideration by ENT practitioners working where there is not possibility to assess the malignancy risk of thyroid nodules by fine needle aspiration biopsy*.
